# The Development of a State-Aware Equipment Maintenance Application Using Sensor Data Ranking Techniques

**DOI:** 10.3390/s20113038

**Published:** 2020-05-27

**Authors:** Haesung Lee, Byungsung Lee

**Affiliations:** Smart Power Distribution Laboratory, KEPCO Research Institute, Daejeon 34056, Korea; bysung@kepco.co.kr

**Keywords:** sensor data, big data, equipment asset maintenance, Internet of Things, state-aware computing, information service, mobile application

## Abstract

Billions of electric equipment are connected to Internet of Things (IoT)-based sensor networks, where they continuously generate a large volume of status information of assets. So, there is a need for state-aware information retrieval technology that can automatically recognize the status of each electric asset and provide the user with timely information suitable for the asset management of electric equipment. In this paper, we investigate state-aware information modeling that specializes in the asset management of electric equipment. With this state-aware information model, we invent a new asset state-aware ranking technique for effective information retrieval for electric power and energy systems. We also derive an information retrieval scenario for IoT in power and energy systems and develop a mobile application prototype. A comparative performance evaluation proves that the proposed technique outperforms the existing information retrieval technique.

## 1. Introduction

Ubiquitous sensing enabled by IoT technologies cuts across many areas of modern-day living. Advances in wireless sensor networks enhance the effectiveness of IoT applications and enrich human life. Among many useful IoT technologies, Paola et al. proposed a modified Stable Election Protocol (SEP), named Prolong-SEP (P-SEP), to prolong the stable period of Fog-supported sensor networks by maintaining balanced energy consumption [1]. The development of such a wireless sensor network technology generates a large amount of sensor data and enables the development of useful IoT applications in human life through intelligent information technology. With the development of ICBM (Internet of Things, Cloud, Big data, Mobile/machine intelligence) technology, which led to the 4th industrial age, Information Retrieval (IR) technology has recently focused on providing users with the information they want on time [2]. 

With the IoT (Internet of Things) in power and energy systems, where a large amount of information is generated at high speed, state-aware computing is considered as a key technology for intelligent information retrieval. Therefore, for the IoT in power and energy systems, unlike the existing computing environment, it is essential to develop state-aware information services that automatically recognize the status of an object and provide appropriate information according to the status of an object.

In the power industry, the Internet of Things (IoT) is at the forefront of this transformation, imparting capabilities such as real-time monitoring, situational awareness and intelligence, control, and cybersecurity to transform the existing electric power energy systems into intelligent, cyber-enabled electric power energy systems. Digitizing the electric power ecosystem using IoT improves asset visibility, optimizes the management of distributed generation, eliminates energy wastage, and creates savings. IoT has a significant impact on electric power energy systems and offers several opportunities for growth and development [3]. Furthermore, in the power industry, the number of electric assets is increasing rapidly, and a tremendous amount of asset information is being intensely generated with the proliferation of electric power IoT [4]. For effective asset management of electric equipment in the IoT of electric power energy systems, it is very important to find only meaningful information from large amounts of asset information and provide the information to decision-makers. In recent years, the importance of asset management in consideration of asset health has been increasing beyond the monitoring and simple diagnosis of assets, so the asset information retrieval application can be effectively used as a basic tool for evaluation of asset health [5]. The health index used to make decisions about maintenance or replacement of equipment is defined based on the status information of the equipment. Furthermore, the accuracy and reliability of the health index can be determined based on what status information of an asset is used [6]. However, in the IoT of electric power energy systems, tremendous amounts of asset information are produced at a high speed, so it is very difficult to find suitable asset status information and use it for effective asset management purposes, such as defining the health index. To overcome these difficulties in the IoT of electric power energy systems and support decision-making for effective asset management, asset information processing technology considering the state of each asset is very necessary. If the status information of an asset can be provided immediately when it is needed, it can significantly reduce the time and cost of processing large amounts of IoT data for asset management decisions. As a result, each asset state awareness technique is of great help for effective asset management, such as defining a more accurate health index of electric equipment. However, due to the enormous amounts of sensor data and the steady increase in equipment assets, studies considering an effective technique for providing equipment status information for asset management are still insufficient. Besides, despite the remarkable development of ICBM technology, there are many difficulties in utilizing sensor data for asset management [7].

In this paper, we propose a technique for asset state-aware information retrieval in the IoT of electric power energy systems that have never been attempted before. The purpose of the proposed technique is to support decision-making for asset management more efficiently. To enable the retrieval of asset information according to the current situation, we define an asset state-aware information model. With the state-aware information model, we invent a new asset state-aware ranking technique for effective equipment asset information services in the IoT of electric power energy systems. The research contribution of this paper is as follows. First, the proposed information retrieval technique enables the development of decision support applications for more effective asset management because it can provide asset information that meets the potential needs of decision-makers. Second, we developed an application prototype that applied the proposed state-aware information technique and demonstrated it based on the actual use scenario. Prototype development and demonstration showed the possibility of using the proposed technique. Lastly, by performing comparative verification, the superiority of the proposed technique was proved, and as a result, it was shown that the proposed technique helped more effectively manage equipment assets in the IoT environment.

This paper is organized as follows. Section 2 discusses context-aware computing in related studies. We also introduce previous studies on information retrieval in IoT environments and discuss their limitations. Section 3 discusses the necessity for state-aware information retrieval in the IoT of electric power energy systems and proposes a state-aware information retrieval technique for equipment based on the sensor data of each electric asset. In Section 4, we implement a prototype of the asset state-aware information retrieval mobile application that specializes in equipment asset management, showing the possibility for practical use of the proposed technique. In Section 5, we compare and verify the existing technique with the proposed technique to show the superiority of the proposed technique. Finally, Section 6 discusses the conclusions and future research.

## 2. Related Works

### 2.1. Context-Aware Computing

Context-aware computing technology, which has been actively researched since the 2000s when the Internet of Things began to emerge, is an information technology that expresses real-world features by combining real-world sensing technology, networking technology, and multimedia technology [8,9,10,11]. Context-aware computing technologies have been applied to the ubiquitous and mobile computing paradigms, playing a key role in the success of these technologies [12,13]. Likewise, the convergence of context-aware computing and sensor technologies will enable the development of various systems: Not only is the amount of data generated over time by a large number of sensors or terminal devices distributed in the power system, but the types of data are also as diverse as the various types of sensors [14]. Therefore, there is a need for an Information Retrieval technique that is specialized for various IoT applications. Alam et al. implemented a context-aware automated cognitive health assessment system, combining the sensing powers of wearable physiological and physical sensors in conjunction with ambient sensors. Based on this system, they developed an automatic cognitive health assessment application in a natural older adult living environment [15]. Eleni et al. focused on the issue of facilitating the management, process, and exchange of the numerous and diverse data points generated in multiple precision farming environments by introducing a framework with a cloud-based context-aware middleware solution as part of a responsive, adaptive, and service-oriented IoT integrated system [16]. In [17], an architecture for building and running context-aware smart classrooms was proposed. The proposed architecture consists of three parts, namely a prototype of a context-aware smart classroom, a model for technology integration, and supporting measures for the operation of smart classrooms in this architecture. 

Schilt and Theimer, who most successfully defined the concept of context awareness, defined contexts as the environment around them or the situation in which they exist [18]. In other words, the context may be referred to as information characterized by the state of an entity existing in the real world. The mean of the entity is an interaction between a human and a thing or things. If information about this interaction can characterize the situation of the object, then that information can be said to be a context [19]. Therefore, context-aware computing may be defined as a computing system that uses the context of an entity in the process of providing appropriate information or services related to a user’s work. Strang et al. classify general context information as follows [20]:

- The context of a user.
- The context of the physical environment.
- The context of a computing system.
- The history of interaction between entities.
- Unclassified situations.

When the classification defined in [20] is applied to the IoT in electric power energy systems, the following context information can be defined:

- Identification information of each electric asset (identifier, installation date, manufacturer, serial number, etc.).
- The sensed state data of each electric asset (temperature, slope, voltage, current, degradation signal, etc.).
- The spatial information (location, direction, etc.).
- Time information (time and season).
- Weather information (temperature, humidity, illuminance, etc.).
- Disaster information (typhoon, heatwave, earthquake, fire, etc.).

The development of asset information service in the IoT of electric power energy systems through the convergence of context-aware computing technology and sensor technology will make the decision-making for asset management of electric assets more effective.

### 2.2. Information Retrieval for IoT 

Information retrieval includes information processing techniques for the presentation of information, the storage of information, the organization of information, and the search for access to specific information to effectively provide the information desired by a user in a short time [21]. Such Information Retrieval technology develops decision-making support services for various types of asset management by finding information that meets the user’s needs from large amounts of information generated from sensors or terminal devices in the IoT environment [22]. However, the intense increase in data due to the expansion of IoT infrastructure in many areas of the world has faced many limitations in finding meaningful information from large amounts of data and making appropriate decisions for asset management. Therefore, the necessity for information retrieval to easily access the required information has been further increased, and thus active research on Information Retrieval technology in the IoT environment is being conducted. Representative studies include [23,24]. Reference [23] proposes an IoT information model specialized for smart building and [24] proposes an IoT standard information retrieval model and indexing technique. The information model proposed by [23] did not consider the characteristics of the unstructured data generated in the IoT environment at all and did not solve the limitation of performance in processing a large amount of sensor data for information retrieval. In turn, [24] proposed an indexing technique specialized for IoT data to improve the efficiency of information retrieval in the IoT environment, but it also cannot consider the characteristics of specific domains, such as the IoT of electric power energy systems, where unstructured data is generated a lot. In the case of the IoT for electric power energy, where the sensor network environment processes and stores unstructured data with different characteristics in the form of information retrieval, users are enabled to easily find the desired thing’s information.

## 3. State-Aware Equipment Maintenance Application Using Sensor Data 

This chapter proposes a new asset state-aware information retrieval technique in the IoT of electric power energy systems. The proposed technique is composed of context-aware computing, sensor techniques, and the Information Retrieval method. 

**Figure 1:**
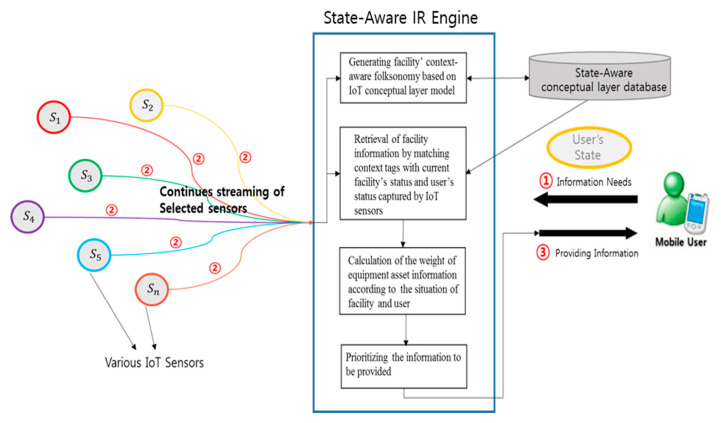
The process of state-aware information retrieval of the Internet of Things (IoT) of electric power energy systems.

Figure 1 shows the conceptual processes of our asset state-aware information retrieval in the IoT of electric power energy systems. As depicted in Figure 1, the asset information tagged with sensor data representing the current state of the equipment asset is stored in a state-aware conceptual layer database. The database stores meaningful data linked to sensor data and equipment information. Looking at the process of providing context-aware information service as shown in Figure 1, first, when a user’s request for information occurs, the situation data of the user is input to the search engine. Then, among the numerous sensor data attached to the equipment, the sensor data that meets the current situation of the user is transmitted as the input value of the information retrieval engine. The state-aware information retrieval engine finally provides information that matches the user’s state and the current state of the equipment based on the state-aware information retrieval method proposed in this paper. With the consideration of asset state, the proposed information retrieval technique enables the development of a decision-support application for more effective asset management because it can provide asset information that meets the potential needs of decision-makers. The proposed information retrieval technique consists of asset state information modeling considering the characteristics of the IoT of electric power energy systems, the automatic asset sensor data tagging technique, and the asset information ranking technique. The proposed sensor data tagging technique is a method of automatically tagging the sensor data to the asset’s information, describing the current state of each asset, as well as providing the information or content of an asset. In other words, by applying the sensor data tagging technique, the real-world information and the digital information can be effectively connected to generate meaningful information that is simultaneously representing the current state of the asset and information related to the asset state. To generate the information tagged with an asset state, we define an asset state-aware information model.

### 3.1. Equipment’s State-Aware Information Model 

**Figure 2:**
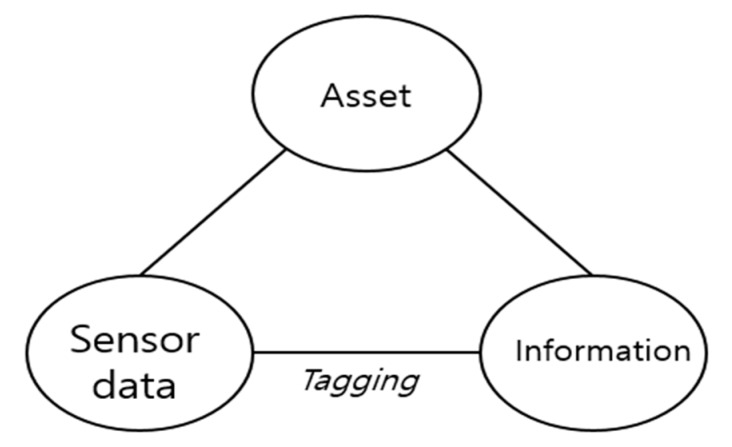
Folksonomy composed of sensor data, an equipment asset, and information.

For the equipment asset’s state-aware information modeling, we organized the equipment asset and the information related to the asset and asset sensor data tag into a folksonomy that has a hyperlink structure, as represented in Figure 2. Folksonomy is a web-based information technology that allows user to upload their resources and to label them with arbitrary words, the so-called tags [25]. Currently, almost all web-based applications provide folksonomy-based information services, and examples of successful applications are Facebook (www.facebook.com), Instagram (www.instagram.com), and YouTube (www.youtube.com). The proposed information model based on folksonomy creates a contextual association between the asset and the information through sensor data annotations. As a result, the asset state-aware information model proposed in this paper makes it easy to generate semantic information that simultaneously represents the current state of the equipment assets and information related to the assets’ state. Information related to a specific asset also can be easily classified by a considered asset state. The use of this classification approach for information suitable for an asset state can be effectively provided for decision-makers who need to manage equipment assets in the IoT of electric power energy systems.

The asset’s state-aware information model in Figure 2 is defined as Definition 1:

**Definition** **1.** *The asset state-aware information model*

- *The asset state-aware information model is represented as a tuple* F:=(A,C,I,R) *, then,*
- *A, C, I are finite sets, where A is the equipment assets, C is the sensor data, and I is information about the equipment asset.*
- *R is a ternary relationship between A, C, and I.*

A folksonomy of the equipment asset generated based on the proposed information model was connected to the other folksonomies and this set of folksonomies has the characteristics of an undirected hypergraph. Through these characteristics, we can define the asset state-aware tagging technique and the equipment asset state-aware information technique that are proposed in this paper.

### 3.2. Equipment’s State-Aware Tagging Algorithm 

The asset state-aware information retrieval technique proposed in this paper includes the automatic asset sensor data tagging technique that considers the asset state based on the information model defined in the previous section. Because power distribution equipment assets are often installed and operated outdoors, these assets are easily broken, or power failures occur due to external environmental factors such as bad weather conditions. Therefore, if the asset state and the asset’s maintenance history information are semantically connected, it is possible to efficiently develop a variety of information services for asset management of the equipment in the IoT of electric power energy systems. Considering these characteristics of assets in the IoT of electric power energy systems, the asset state-aware tagging technique works by deciding whether or not to tag the sensor data with the asset information according to the importance of the situation that has a significant impact on the life of the asset. For example, if a particular equipment asset A is constantly generating abnormal data when it is in situation C, or if the asset A has a lot of asset information related to situation C, it is assumed that situation C has a significant impact on the life of asset A. As a result, the proposed information retrieval technique uses the asset state-aware tagging technique to retrieve the asset information corresponding to both the state of the equipment asset and the user or a decision-maker.
**Algorithm 1** The algorithm for asset state-aware tagging **FOR EACH** (the information set that relates to equipment asset A)**BEGIN****FOR EACH** (the information $D_{j}$ tagged with sensor data $C_{j}$)**BEGIN**${\mathrm{CIW}}_{\mathrm{ij}}$ ← **Divide** the participation frequency of the sensor data $C_{j}$ about the equipment information $D_{j}$ **By** the participation frequency of all tags of sensor data**IF** (${\mathrm{CIW}}_{\mathrm{ij}}>\mathrm{Threshold}$) **THEN** tagging the sensor data $C_{j}$ to information $D_{j}$**End**

Algorithm 1 performs asset state-aware tagging according to the situational importance of the asset state. Firstly, Algorithm 1 calculates the situational importance $CIW_{ij}$. The situational importance $CIW_{ij}$ refers to the influence of the sensor data C on the equipment asset A. The higher the value of $CIW_{ij}$, the more the sensor data C affects the life of asset A. Next, Algorithm 1 extracts the asset information $D_{j}$ that has the value of $CIW_{ij}$ above a certain threshold. Finally, the sensor data $C_{j}$, which consists of situational information, and the value of $CIW_{ij}$ are tagged to the asset information $D_{j}$.

### 3.3. Equipment’s State-Based Information Ranking

In the ranking step of the proposed information retrieval technique, a recommendation weight is given to the asset information in consideration of both the user’s state and the asset’s state to form asset information to be finally provided to the user; that is, the recommendation weight means the degree of matching between the current state of the equipment and the information. Therefore, the higher the weight of information, the greater the degree of conformity to the current state of the equipment. The state-aware information ranking method can effectively provide asset information that matches the user’s state and the current state of the equipment asset. To determine the recommendation weight of the asset information, two factors can be defined as in Definition 2:

**Definition** **2.** *Two factors for calculating the recommendation weight of the asset information,* ${\mathrm{Rank}}_{CD_{a}}$

- $\mathrm{CRC}\left(C_{a},D_{a}\right)$ *means appropriateness of the information* $D_{a}$ *for the sensor data* $C_{a}$ *that negatively impacts the life of an asset A*
- ${\mathrm{CRD}}_{a}$ *means confidence of the information* $D_{a}$ *on asset A*

For effective equipment asset management, it is necessary to recognize the state that negatively affects the life of the equipment and to provide equipment information related to the negative state on time. To recognize conditions that negatively affect facility life and to provide relevant information on time, we defined CRC. To calculate the CRC, we formulate negative state factors that adversely affect asset A, as shown in Equation (1).

(1) $$State_{i}^{negative}=\left(state_{1}^{negative},state_{2}^{negative},\ldots ,state_{n}^{negative}\right)$$

For example, taking into account Equation (1), the set of states that negatively affects the life of the transformer installed in the IoT of electric power energy systems can be defined as follows:

$$Context_{transformer}^{negative}=\left\{context_{transformer}^{temp}:45,context_{transformer}^{oil\_temp}:80,\;context_{transformer}^{uvc}\;:8\right\}$$

Equations (2)–(4) are calculation formulas for calculating the $CRC\left(C_{a},D_{a}\right)$.

(2) $$idf_{c}=log\frac{N}{n_{c}},\;f_{ac_{neg}}=\frac{C_{current}-state_{a}^{negative}}{100}$$

where $idf_{c}$ determines the importance of the sensor data $C_{a}$ for the entire asset information. For $idf_{c}$, N is the number of all asset information and $n_{a}$ is the number of information with which sensor data $C_{a}$ is tagged. $f_{ac_{neg}}$ quantifies the negative impact of the current state $C_{current}$ on the bases of the predefined $state_{a}^{negative}$ for the equipment asset A.

(3) $$w_{dc_{a}}=CIW_{dc_{a}}\times idf_{c},\;w_{dc_{a}^{neg}}=f_{ac_{neg}}\times idf_{c}$$

where $w_{dc_{a}}$ is the weight indicating the importance of the sensor data $C_{a}$ for the asset information $D_{a}$. In $w_{dc_{a}}$, $CIW_{dc_{a}}$ predefined in Algorithm 1 means the importance of the sensor data $C_{a}$ tagged to the asset information $D_{a}$. Furthermore, $w_{dc_{a}^{neg}}$ means the weight for the validity of the asset information $D_{a}$ in the negative state $C_{a}$. $w_{dc_{a}^{neg}}$ is the weight indicating the importance of the asset information $D_{a}$ regarding the negative impact state $C_{a}$ has on the life of equipment asset A. 

Based on Equations (2) and (3), the state suitability $CRC\left(C_{a},D_{a}\right)$ of the asset contents $D_{a}$ for the sensor data $C_{a}$ is calculated through Equation (4).

(4) $$CRC\left(C_{a},D_{a}\right)=\overset{n}{\underset{i=1}{\sum }}w_{dc_{ai}}\times w_{dc_{ai}^{neg}}$$

Next, the formula for calculating $CR_{da}$, which is the reliability of the asset information $D_{a}$ for the asset *A*, is as shown in Equation (5).

(5) $$CR_{da}=\log\frac{wf_{da}}{cf_{da}}$$

In Equation (5), $wf_{da}$ is the number of words in the asset information $D_{a}$ and the $D_{a}$ is the number of times the sensor data $C_{a}$ is tagged in the asset information $D_{a}$; that is, the reliability of the asset information $D_{a}$ with respect to the sensor data $C_{a}$ can be calculated according to the how much information $D_{a}$ contains the contents related to the sensor data $C_{a}$. Finally, the recomend weight $Rank_{CD_{a}}$ of the asset information A is calculatead by summing up the suitablity $CRC\left(C_{a},D_{a}\right)$ of the asset information $D_{a}$ for the sensor data $C_{a}$ and the reliability $CR_{da}$ of the information $D_{a}$ for the sensor data $C_{a}$ and the equipment asset A, as shown in Equation (6).

(6) $$Rank_{CD_{a}}=CRC\left(C_{a},D_{a}\right)+CR_{da}$$

As a result, the rank value of each information quantifies the authority and trust of the contents contained in the information, so that the asset information with high-rank value is more in line with the user’s information request.

## 4. The Development of a State-Aware Equipment Maintenance Application

In this chapter, we implement a prototype of a state-aware equipment maintenance mobile application for IoT in electric power energy systems by applying our proposed technique. The state-aware equipment maintenance application developed in this paper provides useful information that matches the current situation of the equipment around the user’s current location. The developed mobile application helps users to more effectively manage equipment assets by providing users with information on equipment exposed to dangerous situations on time. For the implementation of the application prototype, we consider the following equipment assets:

- cut-out switch;
- pole transformer;
- ground wire;
- insulator;
- wire;
- lightning arrester;
- electric pole.

IoT in electric power energy systems enables the construction of innovative asset management operation models, such as failure prediction, by integrating smart sensor technology into the power grid where equipment assets like transformers, switches, or wires are operated. Considering the characteristics of the IoT of electric power energy systems, we categorized various states that have a negative effect on the equipment asset, as defined as in Table 1, in order to provide timely information suitable for the state that has a relatively high impact on the life of the equipment asset.

Table 2 shows an example of the asset state that can be obtained through smart sensors in the IoT of electric power energy systems, and we considered these states for the development of the prototype application.

The states considered in the prototype can be divided into the equipment asset state and the user’s state, as shown in Table 2. The developed prototype utilizes large amounts of sensor data generated from sensors attached to equipment assets installed on a pole, such as transformers, and thus also any switch in the equipment asset’s state. In addition, the user’s profile data, such as gender, age, and work role, as well as the user’s current location are used as the user’s state in the prototype. These user states are acquired from the IP and GPS of the user’s smartphone. 

**Figure 3:**
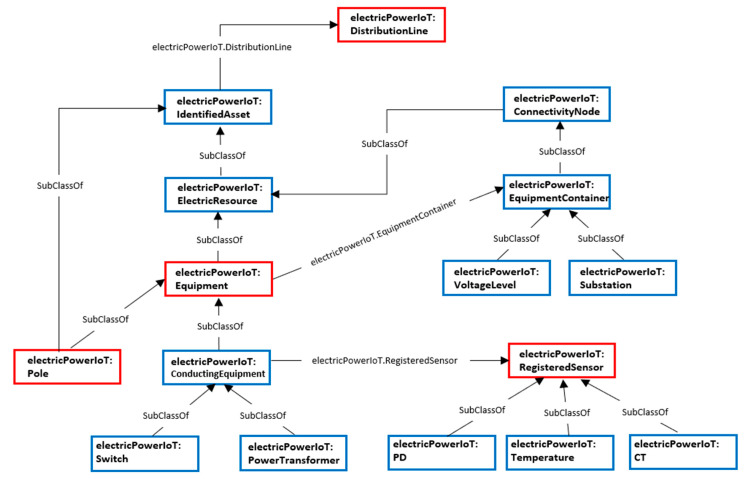
The spatial hierarchy information model used for IoT in electric power energy systems.

Providing large amounts of sensor data, which is one of the limitations of the existing various IoT information services, makes it difficult for the user to access the desired information. To solve this, the implemented prototype restricts the amount of information provided in consideration of the spatial state of the user so that the desired information can be provided more effectively. Figure 3 is part of the spatial conceptual hierarchy model of assets in the IoT of electric power energy systems, based on which the amount of provided information depends on the spatial state of each user. Limiting the amount of provided information according to the spatial state means providing the information according to the semantically hierarchical levels that consider the state of the user and the installed location of the equipment assets.

In other words, unlike general information retrieval services based on user input keywords that provide users with large amounts of unnecessary information, users can obtain the needed information more effectively by showing retrieved information at a limited level by considering the current spatial state.

### 4.1. Implementation Environment and Testing Scenario 

The implemented prototype consists of a server module, a client module, and a state generator, as shown in Table 3. Table 3 shows the implementation environment of each module. The state generator generates the location, weather, and asset state information—like the information obtained from the sensors in the real IoT of electric power energy systems environment—for testing the developed prototype.

**Figure 4:**
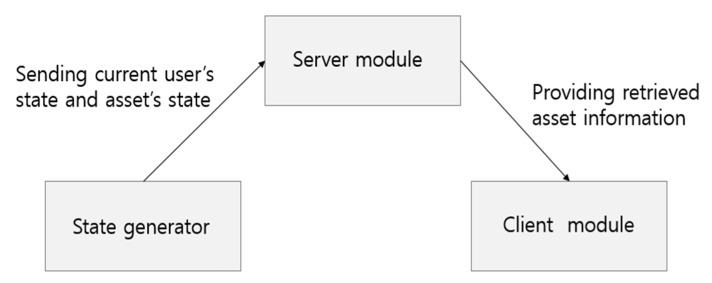
The operation of the developed prototype application.

The operation of the state-aware equipment maintenance application implemented in this paper is shown in Figure 4. The asset state and the user’s state generated from the state generator are transferred to the server module. The server module considers the received sensor data to meet the information need for the user through the asset state-aware information retrieval technique proposed in this paper and provides retrieved asset information through the user’s smartphone.

The service test of our prototype is based on the following scenario. Mr. Lee who owns a GPS-equipped smartphone with the state-aware equipment maintenance application and has a work role of “equipment facilitator”. The state of the season is August and the state of the weather is hot and humid. In addition, the state corresponding to the location of the equipment asset was generated based on the spatial information hierarchical model as shown in Figure 3.

**Figure 5:**
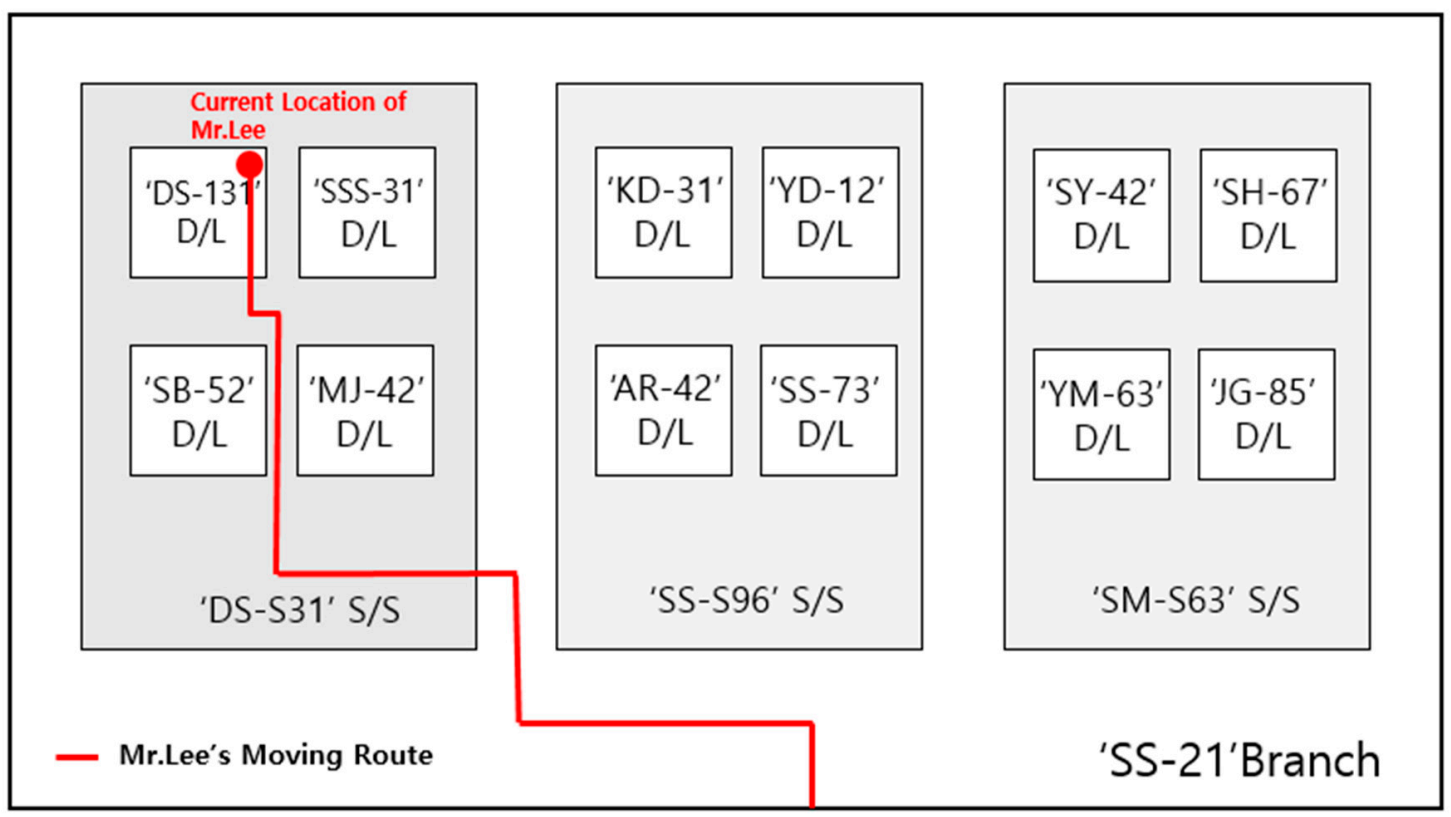
The abstraction map of assets in the IoT of electric power energy systems.

Figure 5 is an abstraction map of the hierarchical inclusion between each location of the S/S (substation), D/L (distribution line), poles, and equipment assets. The moving route of Mr. Lee is also shown in Figure 5. The prototype provides Mr. Lee with asset information that satisfies both the current state of Mr. Lee and equipment assets that are installed in Mr. Lee’s moving route. The implemented prototype will provide Mr. Lee with information about equipment assets that are installed in Mr. Lee’s moving route by considering both Mr. Lee’s state and asset state, based on the defined scenario. In other words, the prototype using the asset state-aware information retrieval technique proposed in this paper provides the user with useful information enabling more effective asset management according to the asset’s state.

### 4.2. Implementation Results 

From the results in Table 4, it can be seen that as the frequency of sensor data tagging increases, the value of the state similarity is larger. In other words, asset information tagged with many specific sensor data can be said to have high reliability. Through this, the automatic sensor data tagging method proposed in this paper has an effective advantage in classifying highly reliable asset information.

**Figure 6:**
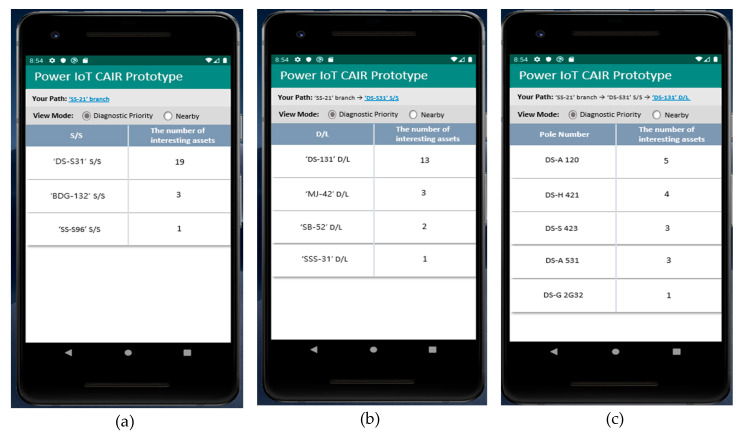
Provision of equipment asset information according to the Mr. Lee’s movement path: (**a**) Mobile app screen when Lee visited the “SS-21” branch; (**b**) mobile app screen when Mr. Lee visited the “DS-S31” substation (S/S); and (**c**) mobile app screen when Mr. Lee visited the “DS-131” distribution line (D/L).

**Figure 7:**
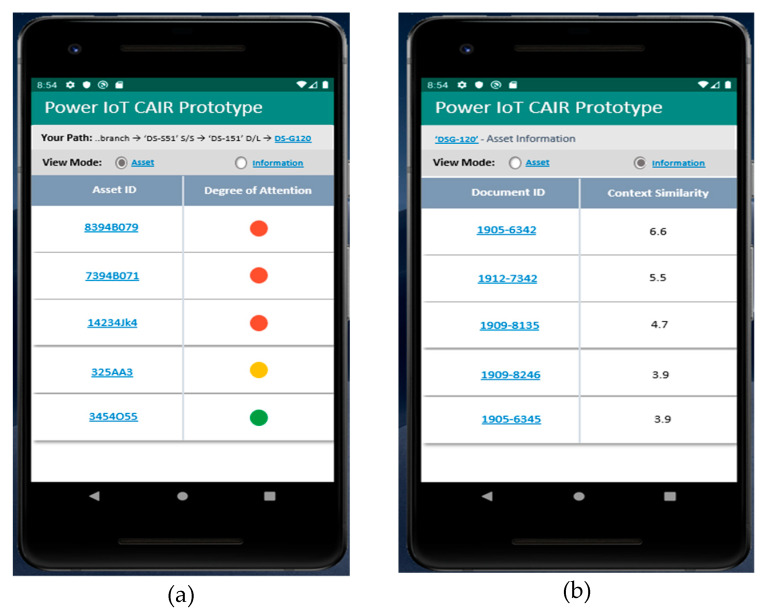
Mobile app screen when Mr. Lee is located near the pole “DS-G120”: (**a**) When the view model is “Asset”, the screen provides interest in the status of the facility asset; (**b**) when the view model is “Asset Information”, the screen provides the facility asset information and information reliability.

Figure 6 and Figure 7 show the operation results of the mobile application prototype implemented by applying the state-aware information retrieval method proposed in this paper.

In general, an information retrieval method returns large amounts of information, including a keyword directly input by a user as a search result. As a result, the existing information retrieval method makes it difficult for users to find the desired information and have limitations in providing reliable information. However, the state-aware information retrieval method proposed in this paper can search for the asset information that matches the user’s state and the asset state without the user’s direct keyword input. As shown in Figure 6 and Figure 7, the information of the equipment in the most negative state among many of the equipment installed close to the user’s current location is given first. Therefore, the developed application prototype can increase the effectiveness of facility asset management by first providing only the information the user needs and increase the user’s satisfaction with the provided information. Through the development of an application prototype, we know that the proposed asset state-aware information retrieval technique can be applied to provide optimal equipment asset information that meets the potential needs of users.

As a result, the developed mobile application based on the state-aware information retrieval technique proposed in the paper helps users to more effectively manage equipment assets by providing users with information on equipment exposed to dangerous situations on time.

## 5. Evaluation

In this section, we perform a comparative performance evaluation between the existing retrieval technique that cannot consider the sensor data and the proposed technique. Through the comparative evaluation, we show that the performance of the asset state-aware information retrieval technique proposed in this paper is superior to that of the existing technique. 

### 5.1. Experimental Design

In constructing the experiment data, 100 documents having a certain number of keyword inclusions and tagged to sensor data tags were randomly generated. Fifty of the documents were classified as “Related” and the remaining 50 were classified as “Not Related”. For calculating the rank value of the documents, with consideration of the relevance of the information, for documents labeled “Related”, we also had 10 documents labeled with “Perfect”, 10 documents labeled with “Excellent”, 10 documents labeled with “Good”, and the remaining 20 documents labeled with “Fair”. Furthermore, to evaluate the performance of the existing search technique, a high-rank value was assigned to a document that has many specific keyword inclusions. The precision technique was used to evaluate the efficacy of the information retrieval systems. Precision is the relationship between the number of retrieved relevant documents R with respect to a query statement Q and the number of documents D that have been retrieved based on it, i.e., R/D [26]. We applied precision as a method to verify the superiority of the proposed technique. In the proposed technique, documents with a high value of state similarity have high-rank values. Based on the experimental data constructed according to the experimental design, the retrieval accuracy of each existing method and the proposed method was calculated through Equation (7).

(7) $$Precision=\overset{N}{\underset{i=1}{\sum }}\frac{RelatedSet\cap Top\left(N_{i}\right)}{RelatedSet}\times \frac{\sum_{i=1}^{N}\left\{Rank\;of\;Top\left(N_{i}\right)\right\}}{N}$$

Equation (7) calculates the search accuracy in consideration of the rank and the relevance to the user’s information request of the retrieved documents that consisted of *Top(N)*.

### 5.2. Precision Performance Result

**Figure 8:**
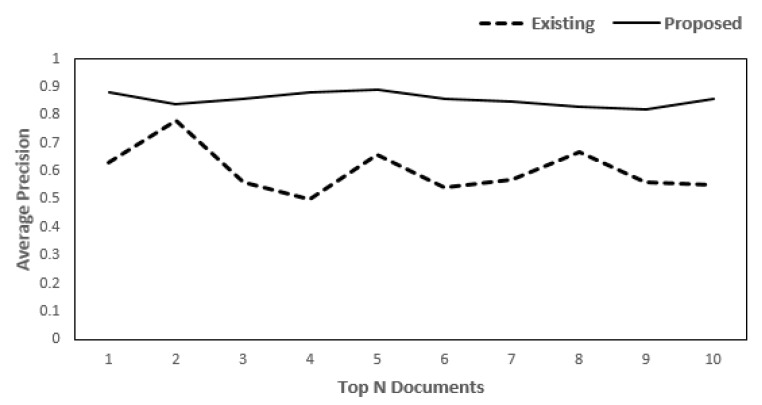
The performance results for the retrieval precision of the existing and proposed techniques.

To compare and evaluate the search performance of the existing and proposed methods, Equation (7) was used to calculate the retrieval precision of the two methods. Figure 8 shows the performance results for the retrieval precision of the existing and proposed methods. As shown in Figure 8, we know that the asset state-aware information retrieval technique outperforms the existing methods. The existing method is based on the traditional information retrieval method. That is, the search relevance of a document is evaluated based on the number of query terms included in the document [27]. In the existing technique, since the relevance and rank of a document are determined only by the number of keywords included in the document, documents that have a large number of keyword inclusions, but do not have an information request, may be retrieved; as a result, as shown in Figure 8, the retrieval precision distribution of the existing technique is not stable. In addition, the existing method provides information including simple keywords without considering the user’s state and the equipment’s state at all, so the search accuracy is lowered and the user satisfaction in providing the information is inevitably reduced. In contrast, because the state similarity of the proposed technique has a decisive influence not only on the rank value of the document but also on the relevance of the document to the user information request, the proposed technique shows a relatively stable distribution of high-precision performance.

## 6. Conclusions

With the IoT in electric power energy systems, tremendous amounts of sensor data are produced at a high speed, so it is very difficult to find suitable asset status information and use it for effective asset management purposes. To overcome these difficulties, we proposed a technique of asset state-aware information retrieval in the IoT of electric power energy systems that have never been attempted before.

To enable the retrieval of asset information according to the current states, we defined an equipment asset state-aware information model. With the information model, we invented a new equipment asset state-aware ranking technique for the development of effective asset information services in the IoT of electric power energy systems. Then, to show the feasibility of our proposed technique, we implemented a prototype of the state-aware information retrieval mobile application for equipment in the IoT of electric power energy systems. The demonstration of the scenario-based prototype showed that the proposed technique is useful for developing information services for effective equipment asset management in the IoT of electric power energy systems. Finally, the comparative performance evaluation of the existing information retrieval method and the proposed technique proved that the performance of the proposed technique is more excellent. In conclusion, the proposed information retrieval technique enables the development of decision-support applications for more effective asset management because it can provide asset information that meets the potential needs of decision-makers.

## Figures and Tables

**Table 1:** The classification of the asset’s negative state.

Classification	Negative State
Defect of facility	Overload, malfunction
Contact of the externals	Trees, birds
Natural phenomenon	Lightning, ice/snow, salt/dust, wind/rain
Natural disaster	Flooding, fire

**Table 2:** An example of acquired sensor data for IoT in electric power energy systems.

Category of States	Location of Sensor Installation		Type of Sensors	Acquired Data
	Main Type	Subtype		
Equipment asset state	Pole	Wire	Partial discharge (PD) smart sensor	The average value of PD
				The average value of fault current
		Transformer	Transformer monitoring smart sensor, Pole current monitoring sensor	Outside temperature
				Internal temperature
				Acceleration
		Cut out switch	Switch smart sensor	Temperature
				Acceleration
User’s state	Mobile phone		IP	User’s identification, User’s profile (Gender, age, work role)
			GPS	Location

**Table 3:** The implementation environment of the prototype.

Modules	Implementation Environment
Server module	Apache Tomcat 9, MySQL, Eclipse, OpenJDK 12, Protégé, Jena, SPARQL
Client module	Android Studio 3.5, OpenJDK 12
State generator	Eclipse, OpenJDK 12

**Table 4:** The partial results of the sensor tagging and the calculation of the state similarity.

Document ID	Asset ID	Pole Number	Frequency of Context Tag				State Similarity
			Defect of Facility	Contact of the Externals	Natural Phenomenon	Natural Disaster	
1912-7342	7394B071	DS-G120	6	5	4	2	5.5
1912-6215	6216C526	KW-S8L77	4	3	2	1	3.4
1911-3216	7416H237	KW-S228	2	3	2	0	2.3
1911-6172	1257J5366	OC-253R4A	4	2	1	0	2.8
1910-7482	8452J4427	DS-H421	1	0	2	2	1.1
1910-7745	9532K647	YG-SSL12	2	3	2	0	2.3
1909-1251	4754T536	DS-S423	4	0	3	2	2.8
1909-6789	5234J0571	DS-G531	1	0	4	3	1.6
1908-8531	6346K536	KW-G517	3	1	1	1	2.1
1908-9626	8342L256	YC-6RA13	2	2	2	2	2.2
1907-3216	9456I536	SDS-H103	1	1	3	2	1.6
1907-8378	4568K478	OC-12T4L	3	0	1	1	1.8
1906-8231	8361L727	YG-SKL5	3	2	5	2	3.3
1906-5884	4756U061	SDG-H1B	2	3	1	1	2.2
1905-4321	2467M461	DS-G2G32	4	1	1	3	2.8
1905-6342	8394B079	DS-G120	7	6	5	3	6.6
1904-1235	5216C526	GW-S8L77	3	2	1	0	2.3
1911-5234	8416H237	GW-S228	3	4	3	1	3.4
1911-7534	2257J5369	OC-253R4A	5	3	2	1	3.9
1910-8757	4452J4423	DS-H421	2	1	3	3	2.2
1910-9245	7532K645	YG-SSL12	1	2	1	0	1.3
1909-8246	9754T537	DS-S423	5	1	4	3	3.9
1909-8135	3234J0578	DS-G531	6	1	5	4	4.7
1908-6482	2346K533	LW-G517	2	0	0	0	1
1908-9734	1342L259	OC-6RA13	3	3	3	1	3.1
1907-8135	9456I537	SDG-H103	0	0	3	3	0.9
1907-8925	8568K472	OC-12T4L	4	1	2	2	2.9
1906-9713	6361L723	YG-SKL5	4	3	4	3	4
1906-9452	5756U068	SDG-H1B	3	2	0	0	2.1
1905-6345	3467M467	DS-G2G32	5	2	2	4	3.9
